# Women’s Experiences and Preferences for Service Delivery of Non-Invasive Prenatal Testing for Aneuploidy in a Public Health Setting: A Mixed Methods Study

**DOI:** 10.1371/journal.pone.0153147

**Published:** 2016-04-05

**Authors:** Celine Lewis, Melissa Hill, Lyn S. Chitty

**Affiliations:** 1 Genetics and Genomic Medicine, UCL Institute of Child Health and Great Ormond Street Hospital for Children NHS Foundation Trust, London, WC1N 1EH, United Kingdom; 2 North East Thames Regional Genetics Service, Great Ormond Street Hospital for Children NHS Foundation Trust, London, WC1N 3BH, United Kingdom; Warwick University, UNITED KINGDOM

## Abstract

Non-invasive prenatal testing (NIPT) for aneuploidy is currently only available in the UK through the private sector outside of the research arena. As part of an implementation study in the UK National Health Service we conducted a mixed methods study to assess women’s experience of being offered NIPT using validated measures of decisional conflict, decisional regret and anxiety. Clinical service preferences were also explored. Women with a Down syndrome screening risk >1:1000 were invited to take part in the study and offered NIPT, NIPT and invasive testing (for women with a risk above 1:150) or no further testing. A cross-sectional survey and semi-structured interviews were conducted at two time points; at the time of testing and one month following receipt of results (or equivalent for NIPT decliners). In total, 845 questionnaires and 81 interviews were analysed. The main motivation to accept NIPT was for reassurance (30.8%). Decisional conflict occurred in a minimal number of cases (3.8%), however, none of the participants experienced decisional regret. Around a third (29.9%) of women had elevated anxiety at the time of testing, including intermediate risk women who traditionally would not be offered further testing (54.4% high risk; 20.1% medium risk), a finding supported through the qualitative interviews where prolonged or additional anxiety was found to occur in some medium risk cases. Women were overwhelmingly positive about the opportunity to have a test that was procedurally safe, accurate, reduced the need for invasive testing and identified cases of Down syndrome that might otherwise have been missed. Reassurance was identified as the main motivator for accepting NIPT, particularly amongst medium risk women, with high risk women inclined to accept NIPT to inform decisions around invasive testing. The current turnaround time for test result was identified as a key limitation. All the women interviewed thought NIPT should be adopted as part of NHS clinical practice, with the majority favouring NIPT offered as a first-line test. Our study highlights the potential that NIPT has to positively impact women’s experience of prenatal testing for aneuploidy.

## Introduction

Screening and diagnostic tests for Down syndrome (DS) have been available for several decades with most Western countries nowadays offering some type of testing. In the UK, the National Screening Committee (UKNSC) recommends that all pregnant women are offered screening for Down syndrome. An information booklet on screening, and the conditions screened for, are given to women before meeting their midwife [1] who will then discuss these issues in more detail. Women accepting DS screening and who book before 14 weeks are offered the combined test which uses a combination of fetal ultrasound, maternal factors and maternal serum biomarkers to determine risk. Those booking after 14 weeks and before 20 weeks are offered the quadruple test which is based on maternal age and serum biomarkers. Women identified as having an increased risk (>1:150) are offered definitive diagnosis with invasive testing however, these tests carry a small risk of miscarriage (0.5%) [2]. These screening tests detect around 85% of DS fetuses for a 5% false positive rate [3]. Prenatal screening and diagnosis is optional and women can decline if they wish.

Prenatal testing is rapidly changing following the development of non-invasive prenatal testing (NIPT) for fetal aneuploidy. NIPT is a blood test which can be used from 10 weeks gestation, is a highly accurate screening test for trisomy 21, 18 and 13 with significantly lower false positive rates and a higher positive predictive value than standard DSS [4–7]. A recent meta-analysis showed detection rates for DS of over 99% with false positive rates of <1% [8]. Due to the small false positive rate NIPT is not considered fully diagnostic and invasive testing is recommended to confirm a positive result [9, 10].

NIPT for DS has been available in the UK through the private sector since 2012, with samples sent to commercial companies in the USA or Hong Kong/China for testing. Research is now underway to assess NIPT uptake when offered through the UK National Health Service (NHS) [11, 12]. Previous psychosocial research exploring hypothetical scenarios showed that women view NIPT as a positive advancement and welcome a test that is safe, accurate and can be conducted early in pregnancy [13–15]. The RAPID (Reliable, Accurate Prenatal, non-Invasive Diagnosis) NIPT Evaluation Study was established to investigate implementation of NIPT into the maternity care pathway in the NHS including psychosocial outcomes relating to patient experience [11]. We have recently established that, given adequate pre-test counselling (whereby women are provided with information about the benefits and risks of NIPT, test accuracy, timings and alternate testing options) and time for reflection, a high proportion of women make an informed choice regarding NIPT for aneuploidy (The Informed Choice study) [16]. In that study we found that 89% of women had made an informed choice; 95% were judged to have good knowledge, 88% had a positive attitude and 92% had deliberated. Here we report on a number of other psychosocial outcomes that were investigated including decisional uncertainty, distress and anxiety, as well as motivations for undergoing or declining NIPT and clinical service preferences. We were particularly interested to see if there were sub-group differences e.g. between different ethnic or education groups, which have been identified elsewhere in the literature, for example, Dormandy et al. identified that South Asian women and socioeconomically disadvantaged women were less likely to make informed choices about DSS [17]. The findings of this study will be useful when developing policy for NIPT implementation in the NHS and other public sector DSS programmes.

## Methods

Approval for this study was obtained from the NHS Research Ethics Committee Camden and Islington (13/LO/0082). Returning a completed questionnaire was taken as implicit consent to take part in the questionnaire study. Interview participants gave either written or verbal consent (if the interview was conducted over the telephone) using an approved consent form. The ethics committee approved this consent procedure.

### Study Design

This was a mixed methods multi-centre study comprising a cross-sectional survey and semi-structured interviews at two time points.

### Sample and Recruitment

A detailed description of the recruitment strategy for the RAPID NIPT Evaluation Study is provided in the study protocol [11]. In summary, NIPT was offered as a contingent screening test to all women with a DSS risk >1:1000 free of charge. Women with a risk >1:150 were also offered the option of invasive testing in line with current practice. All women received face-to-face pre-test counselling with a dedicated NIPT research midwife and written information at two time points (booking-in and pre-test counselling) (S1 Text). NIPT was described as:

- a blood test from the mothers’ arm that was safe for the mother and fetus;
- tests for DS, Edward’s syndrome, Patau syndrome (described as rarer than Down-syndrome and usually life-limiting), and Turner syndrome;
- will detect around 98% of Down’s syndrome cases;
- that possible results include ‘highly unlikely to be affected’, ‘predicted to be affected’, an ‘inconclusive result’ (0.5–4% of cases), or in a small number of cases a ‘failed result’;
- that if the result is ‘predicted to be affected’ an invasive test is needed to confirm the result because in 0.3% of cases NIPT may be incorrect;
- that occasionally there are some rarer chromosomal changes that will not be seen by NIPT but will be seen by invasive testing;
- that results will take 7–10 working days and that testing is voluntary.

In some centres, NIPT was conducted on the same day as DSS and in other they had to return to clinic for the NIPT blood draw. A consecutive sample of women eligible for the Evaluation Study was invited to take part in the Informed Choice study. Whilst acknowledging that non-probability sampling is inherently inferior to probability sampling, this method was chosen as it was most convenient for the recruiters and ensured that recruitment targets for questionnaires were met. Data was collected from eight maternity units located in England and Scotland.

### Questionnaires

This was a longitudinal study in which data was collected at two time points. The first questionnaire (Q1) was given after the blood draw but prior to receiving results (or equivalent for women who declined NIPT). Q1 contained an adapted measure of informed choice [18] as well as The Decisional Conflict Scale, [19] the State Trait Anxiety Inventory (STAI-6), [20] multiple choice questions about women’s reasons for accepting or declining NIPT (informed through previous research) [13, 14], questions around previous pregnancy history and demographics (S2 Table). A second questionnaire (Q2) was either posted or a link to an online version emailed to participants using the online survey website SurveyMonkey (Survey Monkey Inc, Palo Alto, California, USA) one month following receipt of NIPT test results (or equivalent for those that declined testing). Q2 consisted of The Decisional Regret Scale, [21] the STAI-6 [20] and questions about NIPT outcomes (Table 1).

**Table 1:** Summary of measures used in questionnaire.

Measure	Description	Items	Reliability*	Range	Cut-off	Mean (S.D)	Outcome
Decisional conflict scale (Q1)	Measure of uncertainty—low decisional conflict may be viewed as an indicator of informed choice	16 items; 5 point Likert scale (0 = strongly agree– 4 = strongly disagree). Items are summed, divided by 16 and multiplied by 25.	0.96	0–100	≥37.5 indicates decisional uncertainty	9.3 (13.8)	3.8% (n = 22) scored as having decisional uncertainty
Decisional regret scale (Q2)	Measure of distress or remorse after a health care decision	5 items; 5 point Likert scale (0 = strongly agree– 4 = strongly disagree). Items 2 and 4 are reverse coded. Summed scores are multiplied by 25 and divided by 5.	0.75	0–100	No official cut-off, higher scores indicate a higher level of regret	3.2 (7.3)	Results suggest none of the participants experienced decisional regret (0% ≥ 50)
State Trait Anxiety Index, short form (STAI-6) (Q1 & Q2)	Measures state anxiety	6 items; 4 point Likert scale (1 = not at all– 4 = very much). Reverse scoring of items 1,4 and 5. Items are summed, multiplied by 20 and divided by 6.	0.89 (Q1) 0.85 (Q2)	20–80	31–49 considered average. Scores ≥50 indicate elevated state anxiety	Q1: 40.1 (15.5) Q2: 34.3 (12.6)	29.9% (n = 174) scored as having elevated anxiety in Q1. 13.7% (n = 36) scored as having elevated anxiety in Q2.

* Reliability was assessed using Cronbach’s alpha. Scores above 0.7 indicate good internal consistency.

### Interviews

A subset of Q1 responders took part in a telephone interview with CL a week after the NIPT blood draw, or equivalent for NIPT decliners (I1). Women were purposively sampled (maximum variation sampling) to ensure a range of socio-demographic backgrounds, antenatal clinic and testing choices. The interview guide included questions to explore the decision-making process and validate the informed choice measure, questions around motivations for accepting/declining NIPT and perceived benefits and risks of the test. A second set of interviews with CL (I2) were conducted following completion of Q2 around a month after testing (or equivalent for test decliners) with a different subset of women. These interviews focused on participants’ reflections on NIPT and the testing process, and questions around service delivery. In this paper we only report on the qualitative findings around perceived benefits and risks, motivations for accepting or declining NIPT and clinical service preferences. Findings from qualitative analysis focusing on the decision-making process and how women made informed choices around prenatal testing will be presented in a separate paper.

Following consent, interviews were digitally recorded and transcribed verbatim. Data analysis was conducted using thematic analysis [22] and facilitated by Nvivo version 10 software (QSR International, Pty Ltd). Transcripts were read repeatedly and broken down into small meaningful units of texts (codes). For the topics presented in this paper, codes were mostly generated deductively, using the key topics from the interview guide and questionnaire which themselves had been identified through our previous qualitative research in this area [14]. For example, if a participant commented on her desire to remove doubt or fear as her reason for accepting NIPT, it was coded using the equivalent reason cited in the questionnaire if appropriate e.g. for reassurance. Codes were then clustered to form broader categories (motivations for undergoing or declining NIPT and clinical service preferences) in order to answer the aims of the study. Interviews were conducted with women until saturation was reached.

### Data Analysis

Questionnaire data were analysed using SPSS 22 (IBM, Chicago, IL, USA). Reliability of the scales was assessed using Cronbach’s alpha which measures internal consistency [23]. Scores were calculated and dichotomized according to the author’s instructions. Descriptive analysis was conducted on single items, and relationships between categorical variables were examined using chi-squared test, Fisher’s exact test and McNemar’s test for paired data. Qualitative data were analysed using thematic analysis, [22] a method for “identifying, analysing and reporting patterns in the data.” A subset of interviews was also coded by a second researcher (MH) to ensure inter-rater reliability and any discrepancies were discussed until consensus was reached. The interviews from the two time points (I1 and I2) were analysed as a single data set.

## Results

### Sample Characteristics

In total, 731 women were invited to take part in the Informed Choice study and 593 agreed and completed Q1 (81.1% response rate). Eleven questionnaires were subsequently removed due to missing data (N = 582). A summary of maternal characteristics and testing choices are presented in Table 2. For Q2, 536 women agreed to be contacted and 263 responded and were included in the analysis (49% response rate) (Table 3). Q2 responders were significantly more likely to be educated at degree level or above (p = 0.001) and White (p = 0.025) than non-responders. In total, 81 interviews were conducted with participants; 45 at T1, 36 at T2 (75% response rate) and lasted between 12 and 53 minutes. Interview participants’ characteristics and NIPT outcomes are presented as Supporting Information (S1 Table and S2 Table).

**Table 2:** Participant Characteristics.

Participant characteristics	N = 582 n (%)
Maternal age–mean; range	35 years; 19–49
**Educational level**	
No qualification	6 (1%)
GCSE or O level	43 (8%)
GCE, A level or similar	46 (8%)
Vocational (BTEC/NVQ/Diploma)	111 (19%)
Degree level or above	371 (64%)
**Ethnicity**	
White or White British	438 (77%)
Asian or Asian British	61 (11%)
Black or Black British	38 (7%)
Other ethnic group	18 (3%)
Mixed	15 (3%)
**Religious faith**	
Yes	308 (53%)
No	272 (47%)
**Which faith**	
Christian	231 (75%)
Muslim	38 (12%)
Jewish	15 (5%)
Other	8 (3%)
Sikh	7 (2%)
Hindu	6 (2%)
Buddhist	3 (1%)
**Religiosity**	
Very	51 (21%)
Somewhat	162 (66%)
Not at all	33 (13%)
**DSS risk**	
Medium risk	417 (72%)
High risk	165 (28%)
**Further testing**	
NIPT	548 (94%)
NIPT and invasive testing	24 (4%)
No further testing	10 (2%)
**NIPT—same day or different day as DSS**	
Different day	359 (62%)
Same day	196 (34%)
Same day but chose to return	21 (4%)
**Parity**	
Parous	305 (54%)
Nulliparous	267 (47%)
**DSS in previous pregnancy**	
Yes	241 (75%)
No	73 (23%)
Not sure	8 (3%)
**Have a child with DS**	
Yes	1 (<1%)
No	329 (>99)
**Know anyone who has a child with DS**	
Yes	162 (29%)
No	392 (71%)

Note: DSS = Down syndrome screening, DS = Down syndrome, not all % add up to 100 due to rounding. Not all participants answered all questions and therefore there are some discrepancies with total numbers.

**Table 3:** NIPT outcomes.

NIPT outcomes	N = 263 N (%)
**NIPT result**	
Negative	246 (94%)
Positive	10 (4%)
Test failed and did not repeat	4 (2%)
Declined NIPT	2 (<1%)
Inconclusive	1 (<1%)
Action following NIPT result*	
No further testing	233 (97%)
Invasive testing to confirm	7 (3%)
Other	1 (<1%)
Invasive testing result**	
Down syndrome	8 (53%)
Normal result	5 (33%)
T13 or T18	2 (13%)
**Outcome of Down syndrome, T13 and T18 pregnancies**	
Termination of pregnancy	10 (100%)
Continued with pregnancy	0 (0%)

Note: not all % add up to 100 due to rounding. Not all participants answered all questions and therefore there are some discrepancies with total numbers.

*Excludes those women who were high risk and opted for invasive testing at the same time as NIPT

**Includes those women who were high risk and opted for invasive testing at the same time as NIPT

### Questionnaire Results

#### Motivations for accepting or declining NIPT

Table 4 reports women’s motivations for accepting or declining NIPT, most important test attribute and reasons why women declined DSS in previous pregnancies. Reassurance was identified as the main motivator for accepting NIPT (30.8%), particularly amongst medium risk women (26.1% HR, 32.8% MR). ‘To help me make a decision about whether or not to continue with the pregnancy’ was the second most frequently cited motivator for accepting NIPT (20.1%), particularly amongst high risk women (25.4% HR, 17.9% MR). The most frequently cited reason for declining NIPT was feeling sufficiently reassured by the DSS results (30.0%). Safety was identified as the most important factor in the decision to accept NIPT, particularly amongst high risk women (53.1%; HR = 72.7%, MR = 44.9%). Of the 305 parous women in the sample (52.4%), a fifth (20.3%; n = 62) had declined DSS in a previous pregnancy. Reasons why included that it wasn’t offered (25.8%) and they would not have terminated an affected pregnancy (22.6%).

**Table 4:** Motivations for accepting/declining NIPT; most important test attribute for NIPT accepters; reasons why women declined DSS in a previous pregnancy.

Motivations for accepting NIPT	Total N = 910	High riskn = 272	Medium riskn = 638
For reassurance that my baby doesn’t have Down’s syndrome	30.8%	26.1%	32.8%
To help me make a decision about whether or not to continue with the pregnancy	20.1%	25.4%	17.9%
I would want as much information about the baby as possible	19.8%	16.2%	21.3%
Because there is no risk to the baby	9.9%	12.1%	8.9%
So I can plan and prepare for the birth of a baby with Down’s syndrome	9.2%	13.2%	7.5%
To avoid having a child with Down’s syndrome	4.2%	4.0%	4.2%
Because it was offered to me as part of my antenatal care	4.0%	1.1%	5.2%
Other	1.3%	7.4%	1.6%
Because my partner or family would want me to	0.8%	1.1%	0.6%
**Motivations for declining NIPT**	**N = 14**	**n = 1**	**n = 13**
I would never terminate an affected pregnancy so there would be no point taking the test	28.6%	100%	23.1%
I would not want to have to make a decision about whether to terminate the pregnancy	28.6%	0%	30.8%
Feeling sufficiently reassured by DSS results	21.4%	0%	23.1%
It would cause a lot of anxiety if the baby was found to be affected	14.2%	0%	15.4%
My partner or family would not want me to take the test	0%	0%	0%
I would prefer not to know	0%	0%	0%
I chose to go straight for invasive testing	0%	0%	0%
**Most important factor in accepting NIPT**	**N = 524**	**n = 154**	**n = 370**
The safety of the baby (no risk of miscarriage)	53.1%	72.7%	44.9%
Accurate results	30.7%	14.9%	37.3%
Results being available early in pregnancy	9.4%	5.8%	10.8%
The test being freely available	5.2%	4.5%	5.4%
Convenience of the test	1.7%	1.9%	1.6%
Reasons women declined DSS in previous pregnancy*	**N = 33**	**n = 14**	**n = 19**
I would never terminate so there was no point	30.3%	21.4%	36.8%
It wasn’t offered in my previous pregnancy	24.2%	21.4%	26.3%
I would not choose invasive testing and put my pregnancy at risk	18.2%	7.1%	26.3%
Other	9.1%	21.4%	0%
I preferred not to know	6.1%	14.2%	0%
I did not want to know and then have to make a decision about what to do next	6.1%	7.1%	5.3%
It would have caused a lot of anxiety	6.1%	7.1%	5.3%
DSS didn’t give me a definite result	0%	0%	0%
My partner or family did not want me to	0%	0%	0%

Note: N = total number of responses. Participants were allowed to select up to 2 responses for the motivations to accept or decline NIPT and their reason for declining DSS in a previous pregnancy, and only 1 response for the most important test attribute.

*There were 305 parous women in the sample. Of those 62 (20.3%) had declined DSS in a previous pregnancy and 2 declined any further testing in this study. % may not add up to 100 due to rounding.

#### Decisional Conflict and Regret

A cut-off of ≥37.5 is indicated by the authors of the Decisional Conflict measure to indicate decisional conflict or uncertainty [19]. Decisional conflict (Q1) was found to have occurred in a small number of cases (3.8%, n = 22; high n = 5 and medium risk n = 17) at the time of testing. There was no significant association between decisional conflict and DSS results, however there was a significant association between decisional conflict and NIPT uptake with NIPT decliners experiencing significantly more decisional uncertainty than NIPT accepters (20.0% v 3.5%, p = 0.05 Fisher’s Exact). Non-White participants also experienced more decisional uncertainty than White participants (7.8% v 2.4%, p = 0.014 Fisher’s Exact). No formal cut-off for what constitutes decisional regret (Q2) exists, however the scores indicate very low levels of decisional regret (M = 3.17, SD = 7.27) with none of the participants, including those with a positive NIPT result, scoring above the midway point (≥50/100).

#### State-Trait Anxiety Inventory

Around a third of women (29.9%, n = 174) were found to have an elevated state of anxiety at the time of testing (Q1). Unsurprisingly, women with high risk DSS results were found to be significantly more anxious than those with medium risk results (54.5% v 20.1%, X^2^(1) = 66.76, p<0.001), and in the high risk category, women who opted for invasive testing and NIPT concurrently were significantly more anxious than women who opted for NIPT only (73.9% v 51.4%, X^2^(1) = 4.04, p = 0.044). There was no association between anxiety and whether DSS and NIPT counselling took place on the same or different days (p = 0.635). McNemar’s test with continuity correction indicated a significant decrease in anxiety at the time of Q2, (29.9% v 13.7%, X^2^(1) = 24.01, p<0.001). Of the 36 women whose scores indicated elevated anxiety, 30 had a negative NIPT result, 5 had a positive NIPT result (confirmed through invasive testing) and 1 had an inconclusive result (the fetus was found to be unaffected following invasive testing).

### Interview Findings

#### Perceived benefits of NIPT

Women were overwhelmingly positive about the Evaluation Study, valuing the opportunity to have a test that was procedurally safe, accurate, simple to conduct and reduced the need for invasive testing.

*“I would imagine that it was going to dramatically reduce a number of women having to have an amniocentesis* P70, low risk, negative NIPT result

For the vast majority of women, safety was the key attribute of NIPT considered to be most important. A number of women commented that in this respect NIPT was no different to a number of other blood tests they had during pregnancy,

*“There was no risk to the baby at all and it was just a simple procedure that was a blood test*, *because I mean during pregnancy you have lots of them anyway”*P32, low risk, negative NIPT result

Women appreciated that NIPT resulted in cases of DS being identified that might otherwise have been missed:

*“You get told 1 in 30 and although that sounds relatively high…we probably wouldn’t have done [invasive testing] because there’s a risk of miscarriage*…*I think that we were very lucky*. *It’s enabled us to make an informed choice*.*”* P13, high risk, positive NIPT result confirmed through invasive testing and terminated pregnancy

The test also identified an affected pregnancy in a woman who had a medium DSS risk who would traditionally not have been offered further testing:

*“Because we were at a low risk and then all of a sudden it’s high risk*, *that was a shock… but I’m glad I know because I can be prepared*.*”* P27, medium risk, positive NIPT result and continued pregnancy

Many of the perceived benefits of NIPT cited by women were encapsulated in their motivations for accepting NIPT. These are discussed in more detail below.

#### Motivations for accepting or declining NIPT

For women whose DSS risk was medium, the main motivation for accepting NIPT was the opportunity for reassurance and peace of mind. For women whose risk was high, the key motivator was in facilitating decision-making around invasive testing. Women felt “more at ease” basing a decision to undergo “risky” invasive testing based on an NIPT result than on a less accurate DSS result.

*“Had the study not existed*, *I would have been faced with a very difficult decision*, *either not have certainty about the health of the fetus and basically just hope for the best…Or decide to have the invasive test to get certainty which poses quite a high risk to the fetus*.*”* P6, high risk, had NIPT

From a practical perspective, NIPT was described as procedurally easier than invasive testing which required time off work and potentially compromised privacy around the pregnancy. For those women who would not terminate a fetus affected by DS, NIPT offered the opportunity to prepare practically and psychologically and avoid the shock of finding out at the birth. In a small number of cases women declined any further tests. The most common reason cited was because the participant would not terminate an affected pregnancy.

*“I knew I wouldn’t have a termination so I thought that there’s no point going further down and having the extra test*.*”* P14, low risk and declined NIPT

Other reasons cited included that they did not want to return to the clinic for a further blood test, they felt sufficiently reassured by the DSS result, or because the journey to getting pregnant had been so difficult that they would have continued the pregnancy even if the baby had been found to be affected.

Whilst most women identified as high risk following DSS had opted for NIPT, a significant number chose invasive testing, primarily because they did not feel sufficiently reassured by the test accuracy or did not want to wait 7–10 days for the results.

*“It was that waiting period–that being on tenterhooks*, *not knowing*, *and what-have-you*. *It’s awful*.*”* P44, high risk and had NIPT and invasive testing

In a small number of cases, women felt that the indication for aneuploidy was so strong that invasive testing seemed appropriate.

#### Concerns and limitations

In a small number of cases it became apparent that prolonged or additional anxiety had occurred as result of offering medium risk women an additional test.

*“It definitely gave an element of anxiety… If I was treated at another hospital and you just went in and they said ‘you're low risk*, *tick*, *fine’*, *there wouldn't have been any anxiety*, *but we’re still waiting for results…”* P3, medium risk, had NIPT

Nevertheless, the extra reassurance was deemed to “outweigh the anxiety” suggesting prolonged anxiety is considered a reasonable trade-off for a more accurate DSS result.

A number of ethical concerns were raised including a potential increase in termination rates, uneasiness around whether NIPT might result in “eradicating” DS, and whether women would feel pressure to accept NIPT if they had consented to DSS. Regarding views on the limitations of NIPT technology, one pertinent finding related to the implications of a 7–10 working day turnaround for the test results. Three women with a positive NIPT result commented that this then pushed them over the cut-off for having surgical termination as a positive result had to be verified through amniocentesis.

*“I was too late to go through the termination of just taking a pill*. *And that was purely because of the 10 days waiting for the NIPT result … So in other words the standard NHS timings don’t really work if you want to make a decision to not have the baby*, *unless you want to go through a full labour*, *which is pretty horrific*.*”* P8, high risk, positive NIPT result confirmed through invasive testing

Five interviewees had also experienced inconclusive or failed NIPT results and cited these technical issues as limitations which it was important potential users were made aware of prior to testing.

*“I think as long as it’s explained properly what inconclusive means–I think people might worry if someone thought that inconclusive meant that there was likely to be a problem with the baby”*. P73, low risk, inconclusive NIPT result

#### Clinical service delivery

Pre- and post-test counseling: Overall, women were satisfied with the information and support they received during pre-test counselling and felt they had ample time to discuss NIPT and other prenatal testing options with the research midwife. Women felt that NIPT was presented as a clear option, which differed to women’s experience of DSS which some women did not realise was optional.

Participants had a preference for pre and post-test counselling to be conducted by a midwife for reasons including their understanding of the different prenatal testing options and conditions being tested for, because midwives are usually seen for all pregnancy related issues, and for continuity of care.

*“The midwife is very hands on and you get used to seeing them and speaking to them… you kind of draw on their experience a little bit as well*.*”* P55, high risk, had NIPT and invasive testing

Participants also had a preference for NIPT to be conducted the same day as pre-test counselling and ideally on the same day as receiving the DSS results as it was more convenient and did not require returning to the hospital.

Participants had a strong preference for results to be delivered by telephone as this method was quick, convenient, and provided an opportunity to ask questions. If the NIPT result was positive, the importance of being able to come and speak to a health professional as soon as possible was stressed to allow “a face to face chat regarding the possibilities and next steps”. A couple of women thought that patients should be asked how they would like to receive their results, and confirmation of result in a letter was also suggested.

With regards to the terminology used to describe the test results, the vast majority of interviewees were satisfied with ‘highly likely’ or ‘highly unlikely’ as this was considered easier to understand than a risk ratio. Three women had a preference for “a figure” as it was “more tangible”.

#### NIPT as a first-line or contingent screening test

All the women interviewed thought NIPT should be adopted as part of NHS clinical practice with the majority citing a preference for offering NIPT as a first line test replacing DSS. The main reasons cited were that NIPT is more accurate than DSS, that some women would “slip through the net” if it was only offered to women that were high risk, and that offering it to all would enable the majority of women to have reassurance for the remainder of their pregnancy. A number of women also cited the relative ease of interpreting an NIPT result in comparison to a DSS result which “is really complicated to understand.” Notably, it appeared that grappling with two sets of results (DSS and NIPT) created confusion and uncertainty in some cases. One woman who had received a ‘high risk’ screening result and a ‘highly unlikely’ NIPT result said she felt less reassured than if she had only had the NIPT result.

*“I think it’s confusing having two different tests because you're like*, *‘oh do I completely disregard the first round [screening risk of 1 in 12]*?*’”* P16, high risk, had NIPT

Of those women who thought NIPT should be offered as a contingent screening test the main reasons cited were the cost to the NHS, that high risk women would benefit the most, and the potential for NIPT to create additional anxiety for low or medium risk women.

*“If my results were really low*, *one in like two or three thousand*, *I don’t know if I would have gone for it*. *Why would I worry for another two weeks*?*”* P63, medium risk, had NIPT

#### Consent

Most women felt that verbal consent was sufficient for NIPT as there was minimal risk attached to a blood test, it was considered to be similar to screening for which verbal consent suffices, and that written consent might make people apprehensive. Nevertheless, around a third preferred written consent primarily because they believed it would underscore the importance of ensuring that consent for this near-diagnostic test was informed and to protect the hospital against litigation.

## Discussion

This is the first study to assess women’s experience of being offered NIPT as part of the maternity care pathway in a UK NHS setting. Our findings indicate that women were overwhelmingly positive about their experience of NIPT, valuing a test that is safe, accurate and reduces the need for invasive testing. The findings also suggest that more women may take up testing as women who would have previously declined DSS—as they would not have invasive testing due to the risk of miscarriage—would accept NIPT to plan and prepare. Nevertheless, the potential for prolonged or additional anxiety as a result of offering NIPT to women whose DSS risk was medium, and the limitations associated with the current turnaround times were also identified. Despite these concerns, women unanimously agreed that they would like to see NIPT incorporated into the NHS DSS programme.

A key finding from this study was that in around 20% of women in the medium risk group anxiety was elevated whilst waiting for NIPT results. Other studies have also identified elevated anxiety amongst women following an increased DSS risk which reduces on receipt of subsequent reassuring results [24, 25]. For a small minority of women (including both high and medium DSS risk women), anxiety scores were found to remain high even following a negative NIPT result. This residual anxiety might occur for a number of reasons including not having full confidence in NIPT, conflicting messages between NIPT and DSS results, or may reflect elevated baseline anxiety. As we identified, elevated anxiety may be something that women are prepared to tolerate in order to have more information about their pregnancy [26]. However, heightened anxiety in pregnancy has been shown to have potentially harmful effects on the developing fetus [27].

Many factors must be considered when deciding how NIPT should be offered within the NHS DSS pathway. As has been found in other studies, most women advocated offering NIPT as a first line screening test to all women. Similar views have been reported elsewhere amongst parents in the UK as well as Hong Kong [13, 28, 29]. The key advantages of offering NIPT as a first line test are the increased detection of affected pregnancies [30]. However, a recent cost analysis has shown that at its current cost, NIPT is unlikely to be offered as a first line test and is most likely to be offered as a contingent test to women at increased risk [31]. Regardless of how NIPT is incorporated into the DSS pathway, our results suggest that there are a number of issues that should be considered: 1. Women may feel pressured to have NIPT having accepted DSS as they are already part-way down the testing pathway. 2. There is the potential for additional or prolonged anxiety for medium risk women whilst waiting for NIPT results, and 3. two sets of results (DSS and NIPT) may cause confusion for some women. It will therefore be important that health professionals counselling women are well trained and understand the issues that may arise. To mitigate against these problems health professionals should ensure sufficient deliberation is given to the decision to accept NIPT and that it is clear the test is optional; that the accurate turnaround time for the test results is given along with support for women experiencing elevated anxiety during this waiting period; and that understanding of the NIPT results is verified to prevent misunderstandings.

Moreover, we acknowledge that since this study was conducted, some additional limitations of NIPT technology have emerged that may need to be taken into consideration when offering pre-test counselling. This includes that positive predictive values vary significantly depending on prior risk and it has been recommended that this is taken into account when issuing a result. [32].

In this study high-risk (>1:150) women had the option of NIPT or going direct to invasive testing. The 7–10 day turnaround time for an NIPT result and the diagnostic accuracy of invasive testing were key factors in high risk women opting for invasive testing over NIPT. Of note, the majority of women opting for invasive testing had very high DSS risks or sonographic abnormalities. Similar findings have been reported elsewhere [33] suggesting there will still be a significant number of women who prefer invasive testing over NIPT unless the turnaround time notably reduces. A key limitation of the turnaround time for NIPT results related to missing the cut-off for when surgical termination was available if the fetus was found to be affected. Research has shown that, even in the current DSS pathway, NHS services do not fully accommodate choice for women regarding method of termination [34, 35], even though RCOG guidance states that women should be offered a choice [36]. If NIPT is incorporated into NHS practice more widely and the number of tests performed increases, the turnaround time is likely to decrease and so this limitation may be moderated. Finally, an interesting result from our questionnaire was that the most frequently cited reason for declining NIPT was because the responder would not terminate an affected pregnancy which raises the question as to why they opted to have DSS. One possible answer is that these women were unaware that they were undergoing DSS or that screening was optional, findings that has been reported elsewhere in the literature [37].

### Strengths and limitations

A key limitation of this study is the small number of women who declined NIPT. One reason for this is likely to be because NIPT decliners did not return to the clinic and hence the opportunity to recruit them into the study was limited. Nevertheless, only a small number of participants in our study were found to experience decisional uncertainty and none had decisional regret, supporting our previously reported finding that rates of informed choice were high [18]. We also did not include women who declined DSS in this study, although we have conducted previous research with this group [13, 14]. With regards to study design, the study was not conducted with a control group and an intervention group and therefore the results for measures such as anxiety or decisional regret cannot be compared with those not offered NIPT. It could also be argued that the sampling technique used (non-probability) make generalisations from the study to the wider population difficult. A further limitation is the low response rate to Q2 (49%) with non-White responders and responders with lower educational levels more likely to have dropped out. The finding that there was low anxiety and decisional regret a month after testing therefore needs to be treated with some degree of caution as it does not necessarily reflect the experience of certain population groups. The majority of women in our sample were older and well educated, however this may reflect the fact that this group are more likely to be interested in NIPT and are at increased risk as they have delayed childbearing for career purposes. Finally, we acknowledge that the absence of baseline anxiety testing is a drawback as it would have enabled assessment of whether the testing increased anxiety levels or contributed to a reduction for those women whose tests indicated the fetus was unaffected.

## Conclusion

Our study highlights the potential that NIPT has to positively impact intermediate and high risk women’s experience of prenatal testing for aneuploidy. Nevertheless, we have also identified the potential for prolonged or additional anxiety, particularly for intermediate risk women, as a result of the current turnaround time for NIPT results. Care will need to be taken when offering the test to ensure that women fully understand the test procedure and that the benefits of the technology are presented alongside potential limitations and alternate options. Further research to explore why anxiety persists for a subset of women would be useful to determine how post-test counselling could support these women. Further research with NIPT decliners would also be valuable, in particular to explore why they were more likely to experience decisional uncertainty, a finding from this study.

## Supporting Information

S1 Table Interview (I1) participant characteristics. (DOC)

S2 Table Interview (I2) participants NIPT outcomes. (DOC)

S1 Text NIPT for Down Syndrome patient information leaflet. (DOCX)
