# Supplementary material for: Women’s Experiences and Preferences for Service Delivery of Non-Invasive Prenatal Testing for Aneuploidy in a Public Health Setting: A Mixed Methods Study
Source: PLoS One. 2016 Apr 5;11(4):e0153147. doi: 10.1371/journal.pone.0153147 (PMC4821600; doi:10.1371/journal.pone.0153147)
Supplement: S1 Table — (DOC) [file pone.0153147.s001.doc]

S1 Table: Interview (I1) participant characteristics

| **Participant characteristics** | **(n) %** |
| --- | --- |
| Maternal age range | 23-45 |
| **Education** |  |
| No qualification | 0 (0) |
| GCSE or O level | 4 (9) |
| GCE, A level or similar | 3 (7) |
| Vocational (BTEC/NVQ/Diploma) | 6 (13) |
| Degree level or above | 32 (71) |
| **Ethnicity** |  |
| White or White British | 36 (80) |
| Asian or Asian British | 5 (11) |
| Black or Black British | 3 (7) |
| Mixed | 1 (2) |
| **Religion** |  |
| None | 18 (40) |
| Christian | 17 (38) |
| Muslim | 6 (13) |
| Jewish | 3 (7) |
| Hindu | 1 (2) |
| **Parity** |  |
| Nulliparous | 16 (36) |
| Parous | 29 (64) |
| **DSS risk** |  |
| Medium risk | 32 (71) |
| High risk | 213 (29) |
| **NIPT uptake** |  |
| NIPT | 39 (387) |
| NIPT and invasive testing | 3 (7) |
| Declined further testing | 3 (7) |

Note: not all % add up to 100 due to rounding
