# Supplementary material for: Women’s Experiences and Preferences for Service Delivery of Non-Invasive Prenatal Testing for Aneuploidy in a Public Health Setting: A Mixed Methods Study
Source: PLoS One. 2016 Apr 5;11(4):e0153147. doi: 10.1371/journal.pone.0153147 (PMC4821600; doi:10.1371/journal.pone.0153147)
Supplement: S2 Table — (DOC) [file pone.0153147.s002.doc]

S2 Table: Interview (I2) participants NIPT outcomes

| **NIPT outcomes** | **n (%)** |
| --- | --- |
| **NIPT result**  Negative | 24 (67) |
| Positive | 7 (19) |
| Inconclusive followed by negative NIPT result | 3 (8) |
| Test failed and had invasive testing | 1 (3) |
| Test failed and had no further testing | 1 (3) |
| **Action following NIPT result** |  |
| Negative NIPT followed by no further testing | 28 (78) |
| Positive NIPT followed by invasive testing | 6 (17) |
| Positive NIPT followed by no further testing | 1 (3) |
| Negative NIPT followed by invasive testing | 1 (3) |
| **Invasive testing result** |  |
| Down syndrome | 3 (43) |
| T13 or T18 | 3 (43) |
| Normal result | 1 (14) |
| **Outcome** |  |
| Termination of pregnancy | 5 (83) |
| Continued with pregnancy | 1 (17) |

Note: not all % add up to 100 due to rounding
