# Supplementary material for: Women’s Experiences and Preferences for Service Delivery of Non-Invasive Prenatal Testing for Aneuploidy in a Public Health Setting: A Mixed Methods Study
Source: PLoS One. 2016 Apr 5;11(4):e0153147. doi: 10.1371/journal.pone.0153147 (PMC4821600; doi:10.1371/journal.pone.0153147)
Supplement: S1 Text — (DOCX) [file pone.0153147.s003.docx]

NIPT Patient information leaflet Version 4: 15^th^ November 2013

**Non-invasive prenatal testing – a new test for Down’s syndrome**

This leaflet is designed to give you information about a new non-invasive prenatal test (NIPT) for Down’s syndrome. NIPT is much more accurate than the standard screening tests that are available. However, at the moment it is not yet accurate enough to give a definite answer as to whether or not the baby has Down’s syndrome. An invasive test (CVS or amniocentesis) is still necessary to give a definite result. At present NIPT is not available in the NHS and is only being offered to you as part of a research study. For more details about the research please see the participant information sheet. If you are considering using NIPT, please read this leaflet carefully. If anything is unclear, ask your healthcare professional.

**What is Down’s syndrome?**

Down’s syndrome is a life-long condition that causes delays in learning and development, and can cause certain medical problems such as heart problems. It is a variable condition in that some people may be more seriously affected than others. Some adults are able to get jobs and live fairly independent lives; however most people do need long-term help and support. The life expectancy of a person with Down’s syndrome is 60-65 years, although many live longer. Chromosomes are present in almost all human cells and store our genetic information. People usually have 46 chromosomes in each cell, but occasionally extra copies of chromosomes can be present, as is the case in Down’s syndrome. Down’s syndrome is caused by an extra copy of chromosome 21. That is why it is sometimes called trisomy 21. For every 1,000 babies born, one will have Down’s syndrome. Anyone can have a baby with Down’s syndrome, but we do know that the risk increases as women get older. For more information on Down’s syndrome please contact the Down’s syndrome Association. Their contact details are provided at the end of this leaflet.

**What is NIPT for Down’s syndrome?**

We now know that some of the baby’s DNA circulates in the mother’s blood during pregnancy. DNA is the chemical substance that is contained in chromosomes. By looking at the baby’s DNA in the mother’s blood, we are able to identify whether or not the baby is affected by chromosomal conditions like Down’s syndrome. The baby’s DNA is lost from the mother’s blood stream within a few hours of delivery and so testing is specific to the baby in that pregnancy.

**How is NIPT done?**

The test is performed on a sample of the mother’s blood. About 20mls (roughly two tablespoons) is taken from the arm like a normal blood test. The blood is then sent to the laboratory for testing.

**When is NIPT done?**

There is only enough DNA present in the mother’s blood to conduct the test from 10 weeks of pregnancy, so the test cannot be done before then. You will need to have an ultrasound scan first to find out exactly how many weeks pregnant you are and whether there is more than one baby in the womb (such as twins). At present we are not offering NIPT to women with multiple pregnancies.

**How accurate is NIPT for Down’s syndrome?**

NIPT for Down’s syndrome is around 98% accurate. This means that the test detects 98 of 100 cases of Down’s syndrome so there is a very small chance that that the test will not detect an affected pregnancy. In addition there is a small chance (around 0.3%) that the test will incorrectly show that the baby has Down’s syndrome when it does not.

Even though the test is highly accurate there is still the very small chance of an incorrect result. For this reason if you have a predicted to be affected NIPT result you will be offered an invasive test, which is over 99.99% accurate, to confirm the result. Invasive tests are discussed further below.

**How long does it take to get the result?**

It will take about 7-10 working days to get the test result. In 0.5-4% of cases the laboratory is unable to give a result. This might be because there was not enough of the baby’s DNA present in the blood sample to perform the test. If this happens the test can be repeated.

**How safe is NIPT?**

The test is a blood test taken from the arm like a normal blood test. Therefore, the test carries no significant risk to you or your baby. As is the case with all blood tests, there may be some bruising around the area where the blood sample was taken.

**Does NIPT look for conditions other than Down’s syndrome?**

Down’s syndrome is not the only condition that NIPT can look for, although it is the most common. NIPT can also look for rarer chromosome conditions, including Edwards syndrome (trisomy 18) and Patau syndrome (trisomy 13). These conditions are very serious and many affected babies die before or soon after birth. In cases with an increased nuchal translucency measurement (NT 3.5mm) or certain other ultrasound findings the test can also detect Turner syndrome. Like Down’s syndrome, if the NIPT test predicts the baby is affected with any of these conditions an invasive test is recommended to confirm the result.

**How does NIPT compare with other screening tests currently offered during pregnancy?**

The traditional screening test offered during pregnancy, which consists of an ultrasound scan and a blood test (or in some cases only a blood test), is less accurate than NIPT as it only detects 84% - 90% of babies with Down’s syndrome.

**What happens if the NIPT result shows the baby has Down’s, Edwards, Patau or Turner syndrome?**

If the NIPT result shows the baby has one of these chromosomal problems, you will be offered an invasive test. Invasive tests give a definite ‘yes’ or ‘no’ result as to whether the baby has Down’s syndrome. There are two types of invasive test available, an amniocentesis which is usually performed from 15 weeks of pregnancy, and chorionic villus sampling (CVS) which is usually performed between 11 and 13 weeks. Both procedures involve using a fine needle to collect a small sample of either the amniotic fluid that surrounds the baby (amniocentesis) or a small sample of cells from the placenta (CVS). These tests carry a small risk of miscarriage of 0.5% to 1% in the UK. This means that up to 1 to 2 tests in 200 will result in pregnancy loss. Therefore it is important to weigh up how important it is for you to know for certain compared to the risk of miscarriage. If these tests show the baby definitely has Down’s syndrome your options and the management of your pregnancy will be discussed with you.

**Do I have to take the test?**

It is your choice whether or not you take any test in pregnancy, including NIPT. Before making a decision about NIPT, you may want to take some time to consider the test and discuss it with your partner. Think about how you might feel about the test result and how important the information would be for you and your family. If you are unsure about anything, it can be helpful to discuss it with your healthcare professional.

**What happens to the blood sample after NIPT has been done?**

The NIPT samples from women who also had an ultrasound abnormality or NT measurement of ≥3.5mm will be investigated further for other chromosomal rearrangements. No results from these investigations will be available during pregnancy or reported as this is a new area of NIPT development.

Once the test results have been confirmed either by invasive testing or after birth no further testing on the blood is done and the sample will be destroyed.

**Where can I get further information and support?**

These are a list of organisations from whom you can find information and support:

- The Down’s Syndrome Association

A charity supporting people affected by Down’s syndrome.

Telephone: 0333 1212 300

Website: www.downs-syndrome.org.uk

Email: [info@downs-syndrome.org.uk](mailto:info@downs-syndrome.org.uk)

- Antenatal Results and Choices

A charity offering non-directive support and information throughout the antenatal screening and testing process.

Telephone: 0845 077 2290 or 0207 713 7486 from a mobile phone

Website: www.arc-uk.org

Email: info@arc-uk.org

Hours: Monday - Friday: 10am - 5.30pm

These are organisations which provide information about all aspects of pregnancy including antenatal care, screening and testing:

- NHS Choices

www.nhs.uk/conditions/pregnancy-and-baby/pages/antenatal-midwife-care-pregnant.aspx

- National Screening Committee

www.screening.nhs.uk/public
